# Ocular and Plasma Pharmacokinetics of Sitagliptin Eye Drops: Preclinical Data

**DOI:** 10.3390/ph17121579

**Published:** 2024-11-24

**Authors:** Cristina Hernández, Hugo Ramos, Anne Létondor, Rafael Simó

**Affiliations:** 1 Diabetes and Metabolism Research Unit, Vall d’Hebron Research Institute, 08035 Barcelona, Spain; hugo.ramos@vhir.org; 2 Diabetes and Associated Metabolic Diseases Networking Biomedical Research Centre (CIBERDEM), Instituto de Salud Carlos III (ICSIII), 28029 Madrid, Spain; 3 Department of Medicine, Universitat Autònoma de Barcelona, 08193 Barcelona, Spain; 4 Eurofins ADME BIOANALYSES, F-30310 Vergèze, France; anne.letondor@ba.eurofinseu.com

**Keywords:** sitagliptin, dipeptidyl peptidase-4 inhibitor, eye drops, transscleral, pharmacokinetics, diabetic retinopathy

## Abstract

**Background/Objectives:** Early stages of diabetic retinopathy are currently considered an unmet medical need due to the lack of effective treatments beyond proper monitoring and control of glycemia and blood pressure. Sitagliptin eye drops have emerged as a new therapeutic approach against early stages of the disease, as they can prevent its main hallmarks, including both neurodegeneration and microvascular impairment. Interestingly, all of these effects occur without any glycemic systemic improvement. In the present study, we aimed to investigate the pharmacokinetics and distribution of the drug within the eye and plasma. **Methods:** A total of 48 male New Zealand rabbits were treated with topical administration (eye drops) of sitagliptin at two concentrations: 5 mg/mL and 10 mg/mL. Blood, iris/ciliary body, retina/choroid, aqueous humor, and vitreous humor samples were collected at specific intervals post-administration (10 and 30 min and 1, 3, 6, 15, and 24 h), processed, and analyzed using an LC-MS/MS method. The pharmacokinetics of sitagliptin were then calculated, and statistical comparisons were performed. **Results:** Our findings indicate that sitagliptin reaches the retina prior to the aqueous and vitreous humors, suggesting that its absorption follows the transscleral route. Additionally, systemic absorption was minimal and below pharmacologically active concentrations. **Conclusions:** These results support the use of an eye drop formulation for the treatment of diabetic retinopathy and other retinal diseases.

## 1. Introduction

Diabetic retinopathy (DR) is one of the most frequent complications of diabetes mellitus and remains the primary source of blindness among working adults in developed countries [1,2]. Gold standard treatments include laser photocoagulation, intravitreal injections of corticosteroids or anti-VEGF (vascular endothelial growth factor) agents, and vitreoretinal surgery [3]. All of these treatments present limited effectiveness and target the latest stages of the disease, when vision is already compromised. Furthermore, they are invasive and expensive, and they have been associated with secondary severe effects [4]. The treatment of risk factors, such as hyperglycemia, hypertension, and dyslipidemia, is the recommended measure for treating the early stages of DR, but there are no specific treatments to prevent or arrest progression to advanced stages. Therefore, the treatment of early stages of DR is an important unmet medical need.

When treatment is aimed at the initial stages of DR, the use of aggressive routes, such as intravitreal injections, seems out of place, and the use of systemic (i.e., oral route) or topical administration (i.e., eye drops) would be a more appropriate option. However, systemic administration could have serious adverse effects and lead to pharmacologic interferences with other drugs used for the treatment of diabetes and its co-morbidities. In addition, a significant proportion of drugs can hardly reach the retina at pharmacological concentrations due to their limited capacity to cross the blood–retinal barrier. On the other hand, the use of eye drops has not classically been considered a good route for treating retinal diseases. This is because it is generally assumed that they do not reach the posterior segment of the eye (i.e., the retina). In contrast to this pre-established common positioning, recent evidence shows that several peptides/drugs administered through eye drops are able to exert a powerful action in preventing both neurodegeneration and vascular leakage, at least in animal models [5,6,7,8,9,10,11]. Therefore, this implies that they are reaching the retina at pharmacological concentrations. Among them, dipeptidyl peptidase-4 (DPP-4) inhibitors (DPP-4i) seem to be one of the most promising.

In recent years, emerging evidence indicates that the activation of the receptor of glucagon-like peptide-1 (GLP-1R) is an effective therapeutic strategy to treat early DR in experimental models [5,6,10,12,13,14,15]. In this regard, topical administration (eye drops) of native GLP-1, GLP-1 analogues, and DPP-4i has been proven to be effective in preventing the main hallmarks of early stages of the disease (i.e., neurodegeneration and vascular leakage) in experimental models of DR [5,6]. This is not surprising, as GLP-1 has neuroprotective and vasculotropic action. In addition, it should be noted that although both GLP-1R and GLP-1 are synthesized by the retina, GLP-1 production is downregulated by the diabetic milieu. Therefore, the enhancement of GLP-1 directly by using GLP-1 or its analogues or by preventing its degradation by means of DPP-4i can be contemplated as an efficient replacement treatment. However, it should be noted that DPP-4i, in addition to GLP-1R activation, have other mechanisms of action unrelated to GLP-1R activation that can be attributed to other DPP-4 substrates [16,17,18]. In addition, the retinal pigment epithelium (RPE) of subjects with diabetes presents higher DPP-4 concentrations than the RPE of non-diabetic controls matched by age, thus decreasing the availability of GLP-1 for reaching the neuroretina [6]. Furthermore, DPP-4i present higher stability and are cheaper than GLP-1 agonists. All of these properties and recent advances in the understanding of mechanisms of action of sitagliptin (a DPP-4i) make DPP-4i, and, in particular, sitagliptin eye drops, a more promising strategy than GLP-1 or GLP-1 analogues.

In a previous study, we demonstrated that 5 mg/mL was the minimum effective dose showing protective effects of sitagliptin eye drops in a db/db mouse model [14]. In the present study, we have examined the biodistribution and pharmacokinetics of topical administration of sitagliptin in rabbits to cover preclinical regulatory requirements. For this purpose, regulatory agencies recommend the use of rabbits instead of models of diabetic retinopathy. This is because the anatomy of the rabbit’s eye and, in particular, its size are more similar to human eyes than the eyes of mice or rats [19,20].

The results show that sitagliptin eye drops (5 and 10 mg/mL) are rapidly absorbed and that their maximum levels can be found in the iris/ciliary body followed by retina/choroid and the aqueous humor. In contrast, we found practically negligible levels in the vitreous humor, thus suggesting a transscleral route of absorption. Systemic absorption was also very low. These findings support the hypothesis that the effects of sitagliptin eye drops at those concentrations are local and reach the retina very rapidly, thus positioning this treatment as a good candidate to be tested in clinical trials.

## 2. Results

### 2.1. Sitagliptin Levels Among Ocular Tissues

**Figure 1:**
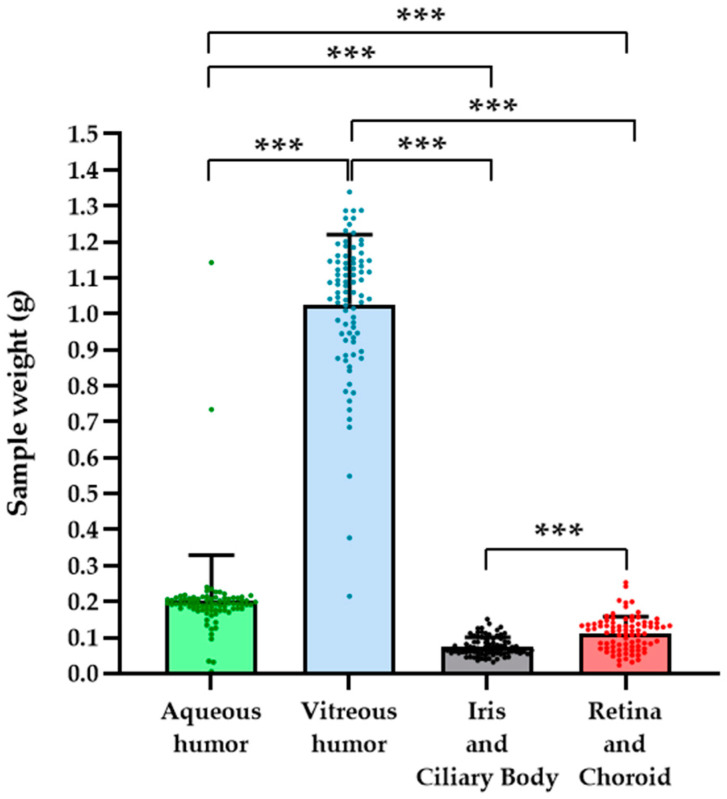
Sample weights. Bar graphs illustrate the mean total sample weight measurements for each eye component studied, independently of eye laterality or treatment received. The green bar represents samples from the aqueous humor, the blue bar represents samples from the vitreous humor, the black bar represents samples from the iris/ciliary body, and the red bar represents samples from the retina/choroid. Individual values are indicated by circle symbols. *n* = 84. *** *p* < 0.001.

First, an exploration of the weights corresponding to the different parts of the eye was carried out in order to detect possible deviations in the values of the calculated concentrations (ng/g tissue) (Figure 1). Regarding sitagliptin distribution through the different ocular tissues, the data and the pharmacokinetic profiles are first represented in a more detailed manner, which implies individual analysis and graphing for each eye (left and right) by averaging the values of the three animals at each extraction time. This was performed separately for each ocular tissue (aqueous humor, vitreous humor, iris/ciliary body, retina/choroid) and for the two tested doses (10 and 5 mg/mL).

**Figure 2:**
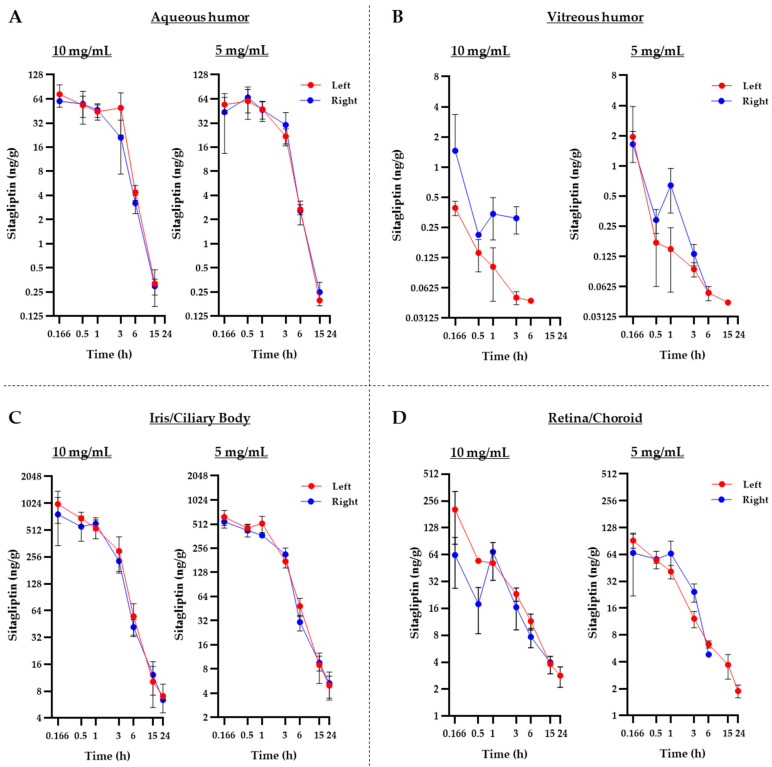
Individual pharmacokinetic analysis of sitagliptin concentrations among the different ocular tissues. (**A**–**D**) Temporary profiles of sitagliptin concentrations in the aqueous humor (**A**), vitreous humor (**B**), iris/ciliary body (**C**), and retina/choroid (**D**) for each dose and eye. A total *n* of 21 rabbits (3 at each time of extraction) was used for each eye. Symbols illustrate the mean value of the 3 animals at each extraction time, while the error bars represent the standard deviation. The concentration and time axes are presented in a logarithmic base-2 scale format.

**Figure 3:**
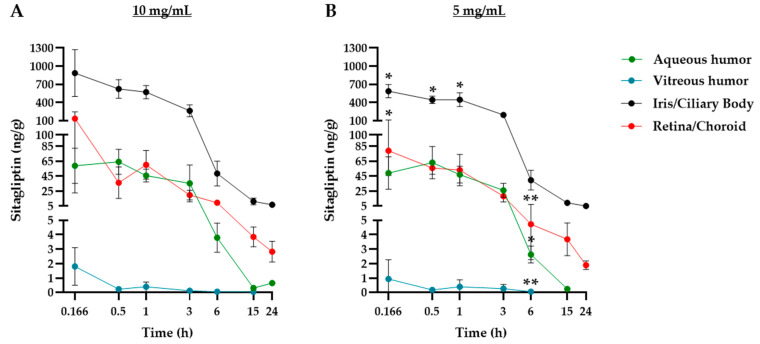
Comparison of sitagliptin pharmacokinetic profiles between the four eye matrices studied. (**A**,**B**) Sitagliptin measurements (ng/g tissue) in the four studied parts of the eye during the experimental course of rabbits treated with sitagliptin 10 mg/mL (**A**) or sitagliptin 5 mg/mL (**B**). The pharmacokinetic profile of the aqueous humor (green circles), vitreous humor (blue circles), iris/ciliary body (black circles), and retina/choroid (red circles). A total *n* of 21 rabbits (3 at each time of extraction) was used per experimental group. The symbols represent the mean of the 3 animals for each extraction time, while the error bars represent the standard deviation. The concentration and time axes are presented in a logarithmic base-2 scale format. The X axis (time) is presented in a logarithmic base-2 scale format. A statistical analysis was conducted to compare the different concentrations, with the time points displaying significant differences highlighted in the graph on the right (5 mg/mL). * *p* < 0.05, ** *p* < 0.01.

Figure 2 depicts the mean profiles of all of these analyses, while Table 1 shows the exact mean values with their respective standard deviations (Figure 2A–D, Table 1). No significant differences were observed between the left and right eyes across all tissues studied. Pharmacokinetic analysis was then performed comparatively between the different tissues in order to determine the degree of exposure to sitagliptin in each of them. The results indicate that the highest drug levels were obtained in the iris and the ciliary body, followed by the retina, the choroid, and the aqueous humor. Finally, the lowest values could be found in the vitreous humor (Figure 3A,B). It is worth mentioning that the results of the vitreous humor reflect a practically negligible level of exposure when compared to the rest of the tissues.

### 2.2. Systemic Absorption of Sitagliptin Eye Drops

**Figure 4:**
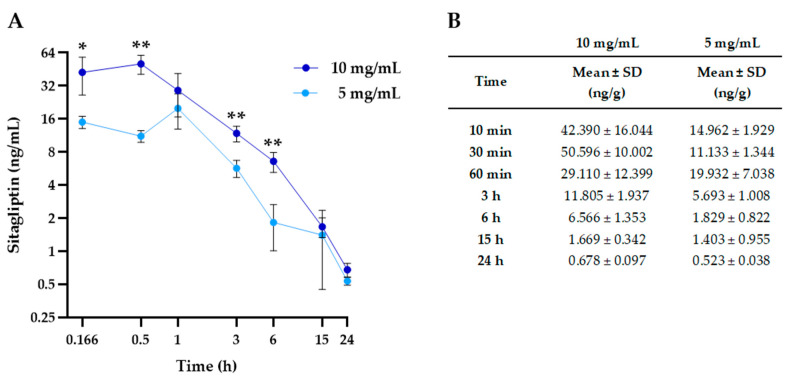
Pharmacokinetic profile of sitagliptin bloodstream levels for both tested concentrations. (**A**) Blood sitagliptin measurements (ng/mL) during the experimental course of rabbits treated with sitagliptin 10 mg/mL (dark blue circles) or sitagliptin 5 mg/mL (light blue circles). A total *n* of 21 rabbits (3 at each time of extraction) was used per experimental group. The symbols represent the mean of the 3 animals for each extraction time, while the error bars represent the standard deviation (SD). The concentration and time axes are presented in a logarithmic base-2 scale format. (**B**) Table exhibiting sitagliptin bloodstream concentrations for both groups during the experimental course. Concentrations are expressed as the mean ± SD of 3 animals at each extraction time. *n* = 21; * *p* < 0.05, ** *p* < 0.01.

In order to evaluate the systemic absorption of both doses of sitagliptin eye drops, one blood sample of each animal was used for the pharmacokinetic analysis. Figure 4A exhibits the pharmacokinetic profile of sitagliptin in the bloodstream, while exact values are displayed in Figure 4B. The observed levels reveal that systemic absorption of sitagliptin is significantly low. Significant differences among the tested doses were found at 10 min, 30 min, 3 h, and 6 h.

Systemic absorption was higher when using a sitagliptin concentration of 10 mg/mL than 5 mg/mL (Figure 4). However, the difference was not so consistent in ocular tissues (Figure 3).

### 2.3. Pharmacokinetic Evaluation of Sitagliptin Eye Drops

The curves illustrating the mean sitagliptin levels that are shown in Figure 2, Figure 3 and Figure 4 were used to perform the pharmacokinetic analysis. Table 2 summarizes the parameters obtained after the analysis. The maximum observed concentration (C_max_) was found in the iris/ciliary body at 10 min (0.166 h) after dose administration compared to the rest of the tissues. The highest levels, or C_max_, and the time at which they were reached, or T_max_, reflect the speed and intensity of drug absorption in that matrix/eye tissue, establishing an absorption flux depending on the tissue. Thus, the iris/ciliary body, the highest, was followed by the retina/choroid and the aqueous humor. The levels observed in the vitreous humor were practically negligible compared to the other tissues.

In parallel, and given that the C_max_ can be correlated with exposure levels between 0 and 24 h (AUC_obs_), the iris/choroid matrix represented approximately 80% of the total. Similar values were obtained for the retina/choroid and the aqueous humor, reaching 10 and 7.5%, respectively. Regarding systemic absorption, the exposure levels were slightly lower in the bloodstream (6%). Finally, total exposure values (AUC_0-∞_) normalized by dose supported a kinetic linearity between both doses (Table 2).

## 3. Discussion

Topical drug formulations based on neuroprotection have successfully been used for treating early stages of DR in rodents, thus suggesting the capability of these drugs to reach the retina at pharmacological concentrations. Because the rabbit eye’s anatomy is more similar to humans in comparison with rodents, this is the required species for pharmacokinetic regulatory purposes [19,20]. The tested doses of sitagliptin eye drops for rabbits were selected following the recommendations of regulatory agencies. When using the ocular topical route, medication is distributed within a small compartment and, therefore, the standard method of converting doses between species based on mg/kg is not appropriate. This method assumes that the drug reaches the target tissue through systemic absorption. To calculate dose conversion between species when using eye drops, the best extrapolation to humans is based on the maximum volume that the conjunctival sac can contain [21]. In this regard, according to eye anatomy, using a topical ocular solution containing a sitagliptin concentration of 5 mg/mL (minimum effective dose in mice) [17], the amount of sitagliptin obtained in the conjunctival sac adjusted by the retinal surface, as well as by the corneal surface, is quite similar in mice and in humans (around 0.50 mcg sitagliptin/mm^2^ for the retina and around 1.2 mcg sitagliptin/mm^2^ for the cornea, respectively).

**Figure 5:**
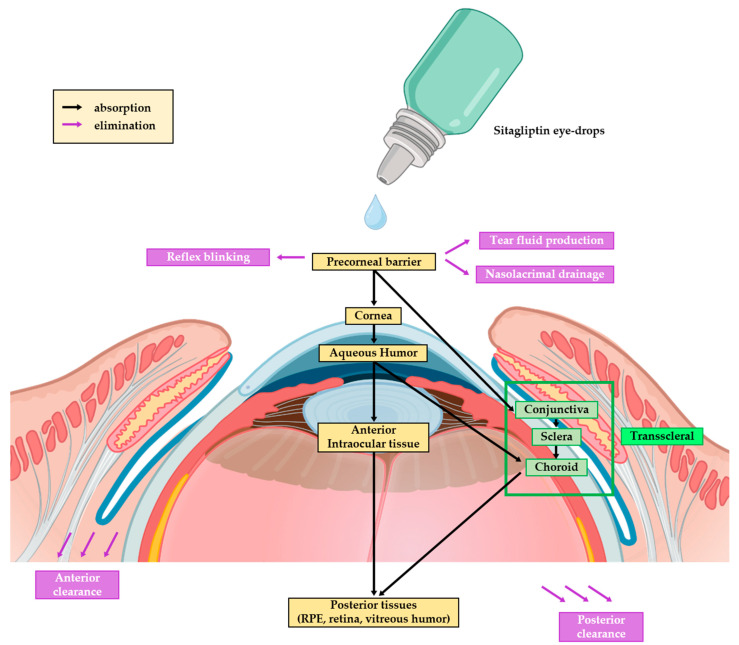
Scheme showing the transscleral route of sitagliptin eye drops’ absorption. After eye drops are applied and distributed across the tear film, the drug is absorbed through the conjunctiva and then penetrates the sclera via diffusion. Certain factors, such as molecular size and lipophilicity, influence this process, with smaller, lipophilic molecules penetrating more easily. Once through the sclera, the drug reaches the choroid and, subsequently, the retina, where it exerts therapeutic effects.

The present study provides evidence that sitagliptin eye drops (5 and 10 mg/mL) administered to rabbits provided quick deliveries to all of the studied eye matrices, and the maximum concentrations were found in the iris/ciliary body, followed by the retina/choroid. In contrast, the concentrations of the vitreous humor were almost negligible. Moreover, the faster delivery and higher AUC observed in the retina/choroid matrix compared to the aqueous humor indicate that the transscleral route is likely playing a more significant role than the traditional transcorneal pathway (Figure 5). This pattern aligns with the behavior of other small and hydrophilic molecules [22,23].

Our pharmacokinetic results can explain the efficiency of topical administration of neuroprotective agents not only in experimental models but also in humans. In this regard, the results of the EUROCONDOR study showed that eye drops of both brimonidine and somatostatin (two neuroprotective agents) prevented the progression of neurodysfunction at the end of follow-up (96 weeks) [24]. This result can hardly be explained by another route to reach the retina than the transscleral route. One can argue about the advantages of topical administration compared to oral or systemic administration of a drug for treatment of DR. The systemic administration of drugs aimed at blocking the main pathogenic pathways involved in DR has two main problems. First, they can hardly reach the retina at pharmacological concentrations. Second, systemic administration could cause serious adverse effects and lead to pharmacologic interferences with other drugs used for the treatment of diabetes and its co-morbidities. In addition, when the early stages of DR are the therapeutic target, it would be inconceivable to recommend an aggressive treatment, such as frequent intravitreal injections [25]. Furthermore, a topical formulation capable of penetrating the retina could reduce systemic side effects and enable patients to self-administer treatments over extended periods [26,27]. For all of these reasons, topical treatment is an emerging new strategy for treating early stages of DR that can revolutionize the management of this devastating complication of diabetes [28].

In the specific case of DPP-4i (i.e., sitagliptin), despite its effect on lowering blood glucose levels by inhibiting GLP-1 degradation, there is no current evidence supporting any beneficial effect in preventing the appearance or arresting the progression of DR when administered orally [29,30,31]. In contrast, as previously mentioned, the beneficial effects of topical administration of these drugs, including, in particular, sitagliptin, have been noted in several experimental studies. This discrepancy can be attributed to fact that this compound cannot cross the blood–retinal barrier.

Regarding safety, DPP-4i have been used as antidiabetic agents for treating type 2 diabetes for more than 15 years and have shown a good safety profile. In the present study, we provide evidence that the systemic distribution of sitagliptin after topical administration is negligible. Systemic absorption may occur through the Schlemm channel or through the uveoscleral outflow connected with the iris. These mechanisms would help to explain the higher levels in the iris/ciliary body matrix and the minimal levels of sitagliptin found in the blood samples [23]. Pharmacokinetic studies of sitagliptin in humans revealed that after administering 100 mg of sitagliptin orally (a commonly used dose for the treatment of type 2 diabetes), a maximum blood concentration of 304 ng/mL of sitagliptin is reached [32]. This concentration is approximately 6 and 15 times higher than the obtained concentrations after administration of sitagliptin eye drops at 10 mg/mL and 5 mg/mL, respectively. Furthermore, oral administrations of 50 mg in humans reached C_max_ ranges between 163 and 182 ng/mL [33]. These values are about 3.5 and 8.6 times higher than those obtained with sitagliptin eye drops at concentrations of 10 mg/mL and 5 mg/mL, respectively. Interestingly, oral administration of 5 mg of sitagliptin in humans, a dose that is within the limit of its hypoglycemic pharmacological activity, reaches a C_max_ similar to the one we obtained after the topical administration of sitagliptin at 10 mg/mL in rabbits [32]. Therefore, the sitagliptin eye drop concentrations that we have tested display minimal systemic distribution without potential interference with any current antidiabetic treatment that can eventually be used in clinical practice.

We have had previous experience with other small molecules administered through eye drops, such as somatostatin, bosentan (a dual antagonist of endothelin receptor), and glucagon-like peptide 1 (GLP-1) [5,11]. All of them showed a pharmacokinetic profile similar to that observed with sitagliptin, thus suggesting that they also reached the retina mainly through the transscleral route. Notably, similar results have recently been reported using anti-VEGF drugs administered through eye drops in rabbits [34]. Thus, bevacizumab eye drops, dosed twice daily, provided higher mean tissue concentrations (ng/g) in the retina, followed by the aqueous humor, the vitreous humor, and the serum. The highest concentration (ng/g) of ranibizumab biosimilar was also present in the retina, followed by the aqueous and vitreous humors. As occurs with sitagliptin, the serum concentration was negligible.

An important point to be examined is whether diabetes itself and age could have any impact on the biodistribution of drugs administered in eye drops. To the best of our knowledge, there are no specific studies on this issue, and it remains a challenge to be met.

Overall, we can conclude that after eye drop administration, sitagliptin reached the retina very rapidly due to intraocular transport through the transscleral route. In addition, its systemic distribution is very low. Therefore, topical administration represents a very promising approach for treating not only diabetic retinopathy but other diseases in which neurodegeneration plays an essential role.

## 4. Materials and Methods

### 4.1. Animals

Forty-eight New Zealand male rabbits (white) were acquired from Granjas San Bernando (Tulebras, Spain) at 12–13 weeks of age and with an initial weight of 2.5–3 kg for the experiments. Animals were randomly distributed (block randomization) into groups of three rabbits per cage under standard laboratory conditions at 15–21 °C, with a relative humidity of 30–70%, 15 air renewals/hour, and a 12 h light/dark cycle. Metallic cages were equipped with ad libitum food (ENVIGO, Mucedola, Milan, Italy) and filtered water.

All animal experiments were directed in agreement with the European Community (86/609/CEE) and the guidelines of the Association for Research in Vision and Ophthalmology (ARVO) for the utilization of laboratory animals. IRB number: DDUNAV 006/23.

### 4.2. Experimental Groups and Topical Ocular Treatment

The 48 animals were distributed into 2 test groups of 21 animals, which were subdivided into 7 subgroups of 3 animals each for the seven different extraction times of the study. Three extra animals were included to obtain basal values (untreated), and another three animals were used as a reserve group to cover possible experimental contingencies. One test group received a topical ocular administration of sitagliptin phosphate monohydrate (SMS Pharmaceuticals Ltd., Hyderabad, Telangana, India) concentrated to 10 mg/mL (1%), while in the other group sitagliptin was concentrated to 5 mg/mL (0.5%). Eye drops were administered only once with the aid of a micropipette onto the superior corneal surfaces of both eyes of each rabbit. The exact volume of eye drops administered to each eye of the animals was 50 µL. Therefore, the amount of sitagliptin administered in each eye drop was 0.25 mg (sitagliptin concentration: 5 mg/mL) and 0.50 mg (sitagliptin concentration: 10 mg/mL).

### 4.3. Anesthesia, Euthanasia, and Sample Processing

The selected extraction times were 10 and 30 min and 1, 3, 6, 15, and 24 h after sitagliptin administration. For the purpose of quantifying the blood levels of sitagliptin at each extraction time and prior to animal euthanasia, blood samples (1 mL) were obtained through the marginal ear vein using lithium heparin tubes and under anesthesia (acepromazine maleate). Sequentially, animals were euthanatized with an injection of a solution composed of embutramide (200 mg), mebezonium iodide (50 mg), and tetracaine hydrochloride (5 mg) (T61, Merck Sharp & Dohme Animal Health, S.L., Salamanca, Spain). Then, the samples of the iris/ciliary body, the retina/choroid, the aqueous humor, and the vitreous humor were acquired. The collection of vitreous and aqueous humor samples was carried out after eye enucleation by using a 25 G needle syringe for the aqueous humor and a sterile Pasteur pipette for the vitreous humor after the cornea and the iris were removed. Ocular tissue samples, specifically the ciliary body and the retina (including the retinal pigment epithelium), along with the choroid, were collected through meticulous dissection using specialized surgical instruments. Each sample obtained from the eye was weighed while fresh and stored in microtubes as independent specimens.

### 4.4. Protein Precipitation and Mass Spectrometry

Extraction prior to quantification was assessed through protein precipitation. In total, 50 µL per sample was isolated in polypropylene tubes for precipitation. Samples were mixed with 10 µL of methanol and 30 µL of acetonitrile (blood, vitreous and aqueous humors) or H_2_O (iris/ciliary body and retina/choroid), and after 30 s of vortex, they were centrifugated at 20,000× *g* and 4 °C for 5 min. Then, supernatants were transferred to polypropylene vials, where 80 µL of H_2_O was also added. Samples were then vortexed again for 30 s and centrifuged at 2500× *g* and 4 °C for 5 min. Sequentially, the levels of sitagliptin were measured through mass spectrometry using an LC-MS 8060 detector (Shimadzu, Kyoto, Japan) and [^2^H_4_]-sitagliptin hemifumarate as the internal standard.

The LC-MS/MS assay was accompanied by the corresponding validation study. The validation process detailed the successful development of the LC-MS/MS method for quantifying sitagliptin in various rabbit eye matrices, including the aqueous humor, the vitreous humor, the ciliary body, the retina/choroid, and whole blood, aimed at preliminary pharmacokinetic studies. Utilizing [^2^H_4_] sitagliptin as an internal standard, the method demonstrated concentration ranges of 0.05 to 10 ng/mL for aqueous and vitreous humors and 0.1 to 20 ng/mL for the ciliary body, the retina/choroid, and whole blood, with sample volumes of 20 μL across all matrices. Extraction techniques included dilution for aqueous and vitreous humors and protein precipitation for the other matrices. The method exhibited excellent precision (intra-run CV generally below 15%), recovery rates ranging from 83.8% to 97.5%, and stability for 2 h at room temperature without any carry-over observed. While the method is robust and versatile for pharmacokinetic investigations, it is important to note that further validation may be required for extensive studies, as only short-term stability was assessed and the impact of potential metabolites or other drugs was not evaluated. Overall, the validation process demonstrated that this LC-MS/MS method provides a reliable tool for quantifying sitagliptin in rabbit models, thus facilitating future research in ocular pharmacokinetics.

The analytical method was previously qualified for eye matrices and blood in a previous study that was further validated according to the ICH M10 guideline [35]. Because protein precipitation exhibited effective analyte recovery and respected the ICH M10 guideline criteria, alternative techniques, such as solid-phase extraction, were deemed unnecessary. Specifically, for sitagliptin, protein precipitation provided highly satisfactory outcomes during development, thus enabling its successful application in both method validation and the analysis of the study samples.

More information on the LC-MS/MS conditions, calibration curves, and quality control is provided in the Supplementary Materials.

### 4.5. Statistical Analysis

Pharmacokinetic characterization of sitagliptin was performed by non-compartmental analysis using the Phoenix WinNonlin Certara^®^ program (version 8.4) [36]. The parameters estimated were the AUC from time 0 to the last experimental point (AUC_obs_), total AUC (AUC_∞_), maximum concentration (C_max_), time at which C_max_ is reached (T_max_) and t_1/2_ of elimination. Differences in ocular and systemic absortion between the 2 concentrations of sitagliptin used (5 and 10 mg/mL) were analyzed by T-Sudent test. A *p*-value < 0.05 was considered statistically significant.

## 5. Patents

Patent PCT/EP2017/060234 addresses the use of dipeptidyl peptidase-4 inhibitors (sitagliptin) for topical eye treatment of retinal neurodegenerative diseases.

## Figures and Tables

**Table 1:** Average levels of sitagliptin over 24 h obtained for each eye and administered dose in the different eye matrices. Data are displayed as mean ± standard deviation (SD) in conjunction with the coefficient of variation (CV). Values under the quantification limit are represented as --. A total *n* of 21 rabbits (3 at each time of extraction) was used for each eye.

	Aqueous Humor							
	10 mg/mL				5 mg/mL			
	Left		Right		Left		Right	
Time	Mean ± SD	CV	Mean ± SD	CV	Mean ± SD	CV	Mean ± SD	CV
	(ng/g)	(%)	(ng/g)	(%)	(ng/g)	(%)	(ng/g)	(%)
10 min	73.13 ± 22.76	31	44.65 ± 16.22	36	54.30 ± 12.47	23	43.85 ± 30.46	70
30 min	53.36 ± 15.80	29	74.74 ± 10.46	14	59.89 ± 24.21	40	66.43 ± 23.54	35
60 min	44.39 ± 9.59	21	47.02 ± 8.98	19	47.42 ± 11.64	24	46.63 ± 13.21	28
3 h	49.47 ± 26.85	54	33.57 ± 8.04	24	21.81 ± 5.24	24	30.31 ± 12.82	42
6 h	4.38 ± 0.93	21	3.20 ± 0.82	25	2.68 ± 0.38	14	2.56 ± 0.85	33
15 h	0.32 ± 0.15	48	0.29 ± 0.06	22	0.13 + 0.06	43	0.25 ± 0.08	32
24 h	0.097 ± 0.009	9	0.28 ± 0.31	109	0.092 + 0.004	4	0.10 ± 0.008	4
	Vitreous Humor							
	10 mg/mL				5 mg/mL			
	Left		Right		Left		Right	
Time	Mean ± SD	CV	Mean ± SD	CV	Mean ± SD	CV	Mean ± SD	CV
	(ng/g)	(%)	(ng/g)	(%)	(ng/g)	(%)	(ng/g)	(%)
10 min	1.95 ± 1.96	101	1.64 ± 0.56	34	0.39 ± 0.06	16	1.46 ± 1.89	129
30 min	0.17 ± 0.11	63	0.29 ± 0.07	27	0.14 ± 0.05	35	0.21 ± 0.01	5.6
60 min	0.15 ± 0.09	63	0.64 ± 0.30	47	0.10 ± 0.05	54	0.67 ± 0.59	87
3 h	0.09 ± 0.01	16	0.13 ± 0.03	24	0.05 ± 0.007	14	0.47 ± 0.29	61
6 h	0.054 ± 0.002	4.5	0.04 ± 0.02	46	0.03 ± 0.015	50	0.02 ± 0.005	22
15 h	0.026 ± 0.014	56	0.02 ± 0.003	18	--	--	--	--
24 h	--	--	--	--	--	--	--	--
	Iris/Ciliary Body							
	10 mg/mL				5 mg/mL			
	Left		Right		Left		Right	
Time	Mean ± SD	CV	Mean ± SD	CV	Mean ± SD	CV	Mean ± SD	CV
	(ng/g)	(%)	(ng/g)	(%)	(ng/g)	(%)	(ng/g)	(%)
10 min	1003.3 ± 394.6	39	766.3 ± 424.0	55	625.7 ± 131.1	21	548.1 ± 90.1	16
30 min	690.1 ± 121.9	18	559.4 ± 177.0	32	457.2 ± 75.2	12	428.2 ± 75.2	18
60 min	537.3 ± 129.5	24	605.3 ± 101.1	17	521.0 ± 120.0	23	369.9 ± 24.0	7
3 h	298.2 ± 129.8	44	230.1 ± 54.2	24	175.6 ± 30.4	17	214.8 ± 42.4	20
6 h	55.2 ± 21.4	39	41.6 ± 9.1	22	48.4 ± 12.4	26	30.6 ± 6.8	22
15 h	10.2 ± 4.9	49	12.1 ± 4.9	40	8.9 + 3.7	41	9.5 ± 2.0	21
24 h	7.1 ± 2.5	35	6.4 ± 0.2	4	5.0 + 1.5	30	5.3 ± 2.0	38
	Retina/Choroid							
	10 mg/mL				5 mg/mL			
	Left		Right		Left		Right	
Time	Mean ± SD	CV	Mean ± SD	CV	Mean ± SD	CV	Mean ± SD	CV
	(ng/g)	(%)	(ng/g)	(%)	(ng/g)	(%)	(ng/g)	(%)
10 min	205.18 ± 121.40	31	63.38 ± 36.46	58	91.37 ± 16.17	17	66.59.1 ± 44.71	67
30 min	54.54 ± 2.07	4	17.87 ± 9.51	53	55.12 ± 4.10	7	56.68 ± 12.60	22
60 min	51.44 ± 18.42	36	68.80 ± 18.81	27	41.04 ± 7.03	17	65.34 ± 24.74	38
3 h	23.16 ± 3.95	17	16.44 ± 7.24	44	12.15 ± 2.53	21	24.25 ± 5.59	23
6 h	11.49 ± 2.32	20	7.66 ± 1.86	24	6.27 ± 0.58	9	4.81 ± 0.06	1
15 h	3.80 ± 0.83	22	2.74 ± 1.11	40	3.68 + 1.14	31	1.62 ± 0.27	17
24 h	2.82 ± 0.72	26	1.63 ± 0.18	11	1.88 + 0.30	16	--	--

**Table 2:** Pharmacokinetic parameters obtained after non-compartmental analysis of the mean sitagliptin levels quantified in the different eye matrices. C_max_, maximum concentration reached; T_max_, time at which C_max_ is reached; AUC_obs_, area under the curve of the concentrations in each matrix from 0 to the last observed point; AUC_∞_, area under the curve of the concentrations in each matrix from 0 to infinity; AUC_∞_/Dose, dose-normalized AUC_∞_. Values under the quantification limit are represented as --. A total *n* of 21 rabbits (3 at each time of extraction) was used for each eye.

	Tissue	C_max_	T_max_	AUC_obs_	AUC_∞_	AUC_∞_/Dose
		(ng/mL)	(h)	(h·ng/mL)	(h·ng/mL)	h·ng/mL/µg
10 mg/mL	Blood	50.596	0.5	155.05	160.05	0.16
	Aqueous Humor	64.051	0.5	201.77	202.57	0.202
	Vitreous Humor	1.799	0.166	1.54	1.73	0.00173
	Iris/Ciliary Body	884.8	0.166	2106.99	2168.39	2.168
	Retina/Choroid	134.28	0.166	251.88	273.94	0.2739
5 mg/mL	Blood	19.93	1	73.46	78.89	0.158
	Aqueous Humor	63.16	0.5	161.2	161.58	0.323
	Vitreous Humor	0.92	0.166	1.31	--	--
	Iris/Ciliary Body	586.9	0.166	1591.26	1636.73	3.273
	Retina/Choroid	78.98	0.166	209.64	240.38	0.480

## Data Availability

The original contributions presented in the study are included in the article/Supplementary Material, further inquiries can be directed to the corresponding authors.

## Supplementary Materials

The following supporting information can be downloaded at https://www.mdpi.com/article/10.3390/ph17121579/s1.
