# Supplementary material for: Ocular and Plasma Pharmacokinetics of Sitagliptin Eye Drops: Preclinical Data
Source: Pharmaceuticals (Basel). 2024 Nov 24;17(12):1579. doi: 10.3390/ph17121579 (PMC11676928; doi:10.3390/ph17121579)
Supplement: Supplementary file 1 [file pharmaceuticals-17-01579-s001.zip › pharmaceuticals-3272287-supplementary.pdf]

Bioanalytical Method

LC-MS/MS CONDITIONS

Mass spectrometer type: Shimadzu 8060
LC/MS/MS quantitation software: Lab solution 6.60
Ionization mode: ESI, positive
Scan Mode:

Column: Kromasil 3x50mm C18
Column Temperature (°C): 40°C
Mobile phase: Mobile Phase A: H20+0.1%formic acid
Mobile Phase B: MeOH +0.1% formic acid

UPLC Gradient :

Time (min)	Flow Rate (mL/min)	A (%)	B (%)
0.00	0.800	90	10
2.00	0.800	10	90
3.00	0.800	10	90
3.10	0.800	90	10
5.00	0.800	90	10

Injection Volume (µL): 1 (AH and VH) 5 (CB and CHR)

Analytes	MS/MS transition	Retention time
2H4 Sitagliptin Hemifumarate	412.2/174.15	1.5
Sitagliptin	408.2/174.15	1.5

Matrix	AH	VH	CB	CHR
Regression	Linear	Linear	Linear	Linear
Weighting	1/X²	1/X²	1/X²	1/X²
LLOQ	2 ng/mL	2 ng/mL	0.05 ng/mL	0.05 ng/mL

Analysis sequence :

Analyst Project name:

	Status	Type of assays	Data .wiff
Run 1	Valid	Assays	
Run 2			
Run 3			
Run 4			

BIOANALYTICAL METHOD

Calibration curve and QC's

Strain of Blank tissues: Matrix provided by the Sponsor
Compound Batch (if different): N/A

Calibration Curve :

AH/VH

Add 5µL of diluted solution in MEOH in 50µL of not diluted blank matrix to obtain calibration standards

Initial spiked solution (µg/mL)	Volume (µL)	Added matrix (µL)	Final concentration matrix (ng/mL)
5.0	**5.00**	**50.0**	500
4.5	**5.00**	**50.0**	450
2.50	**5.00**	**50.0**	250
1.000	**5.00**	**50.0**	100
0.500	**5.00**	**50.0**	50.0
0.100	**5.00**	**50.0**	10.0
0.0400	**5.00**	**50.0**	4.00
0.0200	**5.00**	**50.0**	2.00

CB/CHR

Add 5µL of diluted solution in MEOH in 50µL of diluted blank matrix (1/5 in ACN) to obtain calibration standards

Initial spiked solution (µg/mL)	Volume (µL)	Added matrix (µL)	Final concentration matrix (ng/mL)
0.1	**5.00**	**50.0**	10
0.09	**5.00**	**50.0**	9
0.05	**5.00**	**50.0**	5
0.030	**5.00**	**50.0**	3
0.010	**5.00**	**50.0**	1.0
0.005	**5.00**	**50.0**	0.5
0.0010	**5.00**	**50.0**	0.10
0.0005	**5.00**	**50.0**	0.05

Quality Controls :

Add 5µL of diluted solution in MeOH in 50 µL of not diluted blank matrix to obtain QC samples

Initial spiked solution (µg/mL)	Volume (µL)	Added matrix (µL)	Final concentration matrix (ng/mL)
4.0	**5.00**	**50.0**	400
2.5	**5.00**	**50.0**	250
0.060	**5.00**	**50.0**	6.0

Add 5µL of diluted solution in MeOH in 50 µL of not diluted blank matrix to obtain QC samples

Initial spiked solution (µg/mL)	Volume (µL)	Added matrix (µL)	Final concentration matrix (ng/mL)
0.08	**5.00**	**50.0**	8
0.05	**5.00**	**50.0**	5
0.002	**5.00**	**50.0**	0.2

Acceptance criteria : 6 calibration levels and 75% of all calibration standards must fall within ± 20.0% of nominal (± 25.0% at the LLOQ)
66.6 % of QCs must fall within ± 20% of nominal and minimum one QC accepted per level and block of QCs

Extraction Method: **Protein Precipitation**

AH/VH

Sample Volume: 20 µl of sample
Sample Preparation: Add 2µL MeOH and vortex
Add 10 µL of 1 µg/mL ISTD (Sitagliptin hemifumarate) in MeOH
Add 30 µL of **ACN**
Centrifuge at 20 000g and 4°C for 5 minutes
Transfer in polypropylen vials
Centrifuge at 2 500g and 4°C for 5 minutes then inject in LC-MSMS

CB/CHR

Sample Volume: 20 µL of sample extract (1 CHR or 1 CB + 5 mL ACN for 3 h under agitation)
Add 2 µL of MeOH and vortex
Add 10 µL of 0.05 µg/mL ISTD (Sitagliptin hemifumarate) in MeOH
Add 30 µL of **H2O**
Centrifuge at 20 000g and 4°C for 5 minutes
Transfer in polypropylen vials
Centrifuge at 2 500g and 4°C for 5 minutes then inject in LC-MSMS
